# P-2320. Clinical Significance of Colonization with Fluoroquinolone-Resistant Enterobacterales in Hematopoietic Cell Transplant Recipients and Patients with Acute Leukemia

**DOI:** 10.1093/ofid/ofae631.2472

**Published:** 2025-01-29

**Authors:** Jamie Marino, Jessica Oxer, Catherine Liu, Alison Freifeld, Lijuan Zeng, Lauren Komarow, Sarah B Doernberg, Scott D Rowley, Ryan K Shields, Samuel A Shelburne, Morgan Hakki, David van Duin, Samantha E Jacobs, Liang Chen, Barry N Kreiswirth, Thomas J Walsh, Henry Chambers, Vance G Fowler, Lars Westblade, Michael J Satlin

**Affiliations:** Weill Cornell Medicine, NY, New York; Weill Cornell Medicine, NY, New York; Fred Hutchinson Cancer Center, Seattle, WA; University of Nebraska Medical Center, Omaha, NE; The Biostatistics Center, George Washington University, Washington, District of Columbia; George Washington University, Rockville, Maryland; University of California, San Francisco, San Francisco, CA; Georgetown University School of Medicine, Fernandina Beach, Florida; University of Pittsburgh, Pittsburgh, Pennsylvania; MD Anderson-University of Texas, Houston,, Texas; Oregon Health and Science University, Portland, OR; University of North Carolina at Chapel Hill, Chapel Hill, NC; Icahn School of Medicine at Mount Sinai, New York, New York; University of Buffalo, Buffalo, New York; Center for Discovery and Innovation, Hakensack Meridian Health, Nutley, New Jersey; Center for Innovative Therapeutics and Diagnostics (citdx.org), Richmond, VA; University of California San Francisco, San Francisco, California; Duke University Medical Center, Durham, NC; Weill Cornell Medicine, NY, New York; Weill Cornell Medicine, NY, New York

## Abstract

**Background:**

Hematopoietic cell transplant (HCT) recipients and patients with acute leukemia are at high risk of Gram-negative bacteremia during neutropenia. Fluoroquinolone (FQ) prophylaxis is used to prevent bacterial infections during neutropenia, but the impact of colonization with FQ-resistant Enterobacterales (FQRE) on its effectiveness is unclear.

**Figure:**
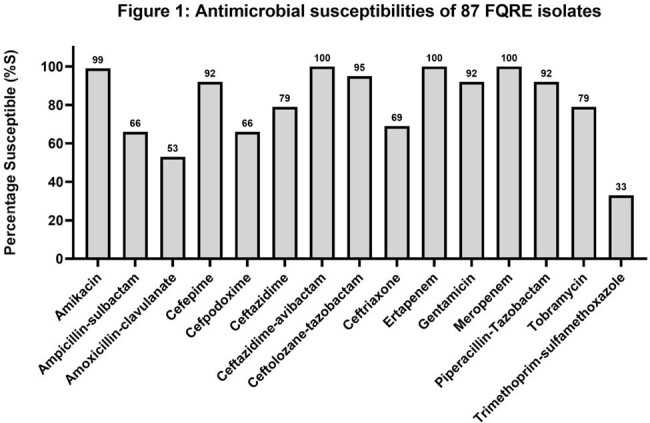

**Methods:**

Adult patients undergoing HCT or receiving induction chemotherapy for acute leukemia were enrolled at 10 U.S. centers from 2021 to 2023. Patients received FQ prophylaxis during neutropenia. Perianal swabs were collected within 4 days of initiating chemotherapy or during the week before HCT, and underwent selective broth enrichment culture for FQRE. Isolates underwent antimicrobial susceptibility testing by disk diffusion. Risk factors for FQRE colonization were identified and infections prior to neutrophil recovery were compared between FQRE-colonized and non-colonized patients.

**Figure:**
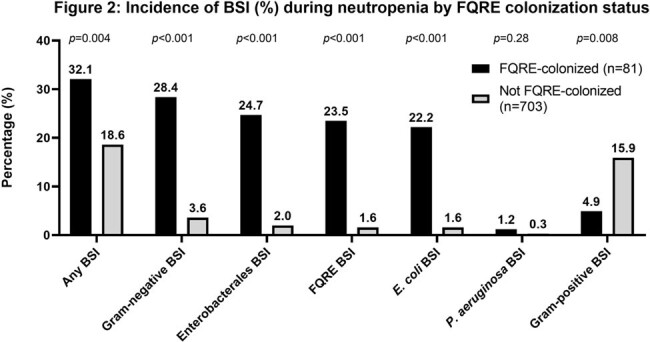

**Results:**

Among 784 patients (376 allogeneic HCT recipients, 291 autologous HCT recipients, and 117 receiving chemotherapy for acute leukemia), 81 (10.3%) were colonized with FQRE. Risk factors for FQRE colonization were non-White race, prior HCT, receipt of a β-lactam or any antibacterial agent within last 3 months, and having prior detection of FQRE or a 3^rd^-generation cephalosporin-resistant Enterobacterales (Table 1). Of the 87 colonizing FQRE isolates (*Escherichia coli*: n=71; *Klebsiella pneumoniae*: n=12), 33% were susceptible to trimethoprim-sulfamethoxazole, 53% to amoxicillin-clavulanate, and 66% to cefpodoxime (Figure 1). FQRE-colonized patients had increased risk of bloodstream infections (BSIs) due to any Gram-negative bacteria (28.4% vs. 3.6%; *p*< 0.001) and FQRE (23.5% vs. 1.6%; *p*< 0.001) compared to non-colonized patients (Figure 2). They also had an increased risk of infections other than BSIs (12.3% vs. 4.0%; *p*=0.003) and intensive care unit admissions (12.3% vs. 4.2%; *p*=0.005) prior to neutrophil recovery, but similar 90-day mortality (4.9% vs. 4.0%, *p*=0.6).

**Conclusion:**

Screening for FQRE colonization identifies patients at high risk of Gram-negative bacteremia following FQ prophylaxis. Alternate infection prevention strategies are needed in FQRE-colonized patients.

**Disclosures:**

Catherine Liu, MD, Pfizer: Grant/Research Support Sarah B. Doernberg, MD, MAS, Basilea Pharmaceutica: Grant/Research Support|F2G Limited: Grant/Research Support|Genentech: Advisor/Consultant|Gilead Biosciences: Grant/Research Support|Janssen/J+J: Advisor/Consultant|Pfizer, Inc: Grant/Research Support|Regeneron, Inc: Grant/Research Support|Shinogi: Grant/Research Support Scott D. Rowley, MD, COTA: Stocks/Bonds (Private Company)|Genetic Testing Cooperative: Stocks/Bonds (Private Company)|SirPant Immunotherapeutics: Advisor/Consultant Ryan K. Shields, PharmD, MS, Allergan: Advisor/Consultant|Cidara: Advisor/Consultant|Entasis: Advisor/Consultant|GSK: Advisor/Consultant|Melinta: Advisor/Consultant|Melinta: Grant/Research Support|Menarini: Advisor/Consultant|Merck: Advisor/Consultant|Merck: Grant/Research Support|Pfizer: Advisor/Consultant|Roche: Grant/Research Support|Shionogi: Advisor/Consultant|Shionogi: Grant/Research Support|Utility: Advisor/Consultant|Venatorx: Advisor/Consultant|Venatorx: Grant/Research Support David van Duin, MD, PhD, Merck: Advisor/Consultant|Merck: Grant/Research Support|Pfizer: Advisor/Consultant|Qpex: Advisor/Consultant|Roche: Advisor/Consultant|Shionogi: Advisor/Consultant|Shionogi: Grant/Research Support Samantha E. Jacobs, MD, MS, Ansun Biopharma: Advisor/Consultant|Eurofins, Viracor, LLC.: Grant/Research Support Thomas J. Walsh, MD, PhD (Hon), FIDSA, FAAM, FECMM, Abbott Laboratories: Advisor/Consultant|Basilea: Advisor/Consultant|Basilea: Grant/Research Support|Cape Cod Associates: Advisor/Consultant|F2G: Advisor/Consultant|F2G: Grant/Research Support|Omeros: Advisor/Consultant|Omeros: Grant/Research Support|Partner Therapeutics: Advisor/Consultant|Scynexis: Advisor/Consultant|Scynexis: Grant/Research Support|Statera: Advisor/Consultant|T2 Biosystems: Advisor/Consultant|T2 Biosystems: Grant/Research Support Henry Chambers, MD, Merck: Stocks/Bonds (Private Company)|Moderna: Stocks/Bonds (Private Company) Vance G. Fowler, MD, MHS, Affinergy: Advisor/Consultant|ArcBio: Stocks/Bonds (Private Company)|Armata: Advisor/Consultant|Astra Zeneca: Advisor/Consultant|Astra Zeneca: Grant/Research Support|Basilea: Advisor/Consultant|Basilea: Grant/Research Support|ContraFect: Advisor/Consultant|ContraFect: Grant/Research Support|Debiopharm: Advisor/Consultant|Destiny: Advisor/Consultant|EDE: Grant/Research Support|Genentech: Advisor/Consultant|Genentech: Grant/Research Support|GSK: Advisor/Consultant|Janssen: Advisor/Consultant|Karius: Grant/Research Support|MedImmune: Grant/Research Support|Merck: Grant/Research Support|sepsis diagnostics: Patent pending|UptoDate: Royalties|Valanbuio: Stocks/Bonds (Private Company)|Valanbuio: Stocks/Bonds (Private Company) Lars Westblade, PhD, Accelerate Diagnostics, Inc: Grant/Research Support|bioMerieux, Inc: Grant/Research Support|Element Materials Technology: Grant/Research Support|Hardy Diagnostics: Grant/Research Support|Roche Molecular Systems, Inc.: Advisor/Consultant|Roche Molecular Systems, Inc.: Grant/Research Support|Selux Diagnostics, Inc.: Grant/Research Support|Shionogi, Inc: Advisor/Consultant|Talis Biomedical: Advisor/Consultant Michael J. Satlin, MD, AbbVie: DSMB participant|bioMerieux: Grant/Research Support|Merck: Grant/Research Support|Selux Diagnostics: Grant/Research Support|SNIPRBiome: Grant/Research Support

